# Volume-based solvation models out-perform area-based models in combined studies of wild-type and mutated protein-protein interfaces

**DOI:** 10.1186/1471-2105-9-448

**Published:** 2008-10-21

**Authors:** Salim Bougouffa, Jim Warwicker

**Affiliations:** 1 Faculty of Life Sciences, the Michael Smith Building, University of Manchester, Oxford Road, Manchester, M13 9PT, UK

## Abstract

**Background:**

Empirical binding models have previously been investigated for the energetics of protein complexation (ΔG models) and for the influence of mutations on complexation (i.e. differences between wild-type and mutant complexes, ΔΔG models). We construct binding models to directly compare these processes, which have generally been studied separately.

**Results:**

Although reasonable fit models were found for both ΔG and ΔΔG cases, they differ substantially. In a dataset curated for the absence of mainchain rearrangement upon binding, non-polar area burial is a major determinant of ΔG models. However this ΔG model does not fit well to the data for binding differences upon mutation. Burial of non-polar area is weighted down in fitting of ΔΔG models. These calculations were made with no repacking of sidechains upon complexation, and only minimal packing upon mutation. We investigated the consequences of more extensive packing changes with a modified mean-field packing scheme. Rather than emphasising solvent exposure with relatively extended sidechains, rotamers are selected that exhibit maximal packing with protein. This provides solvent accessible areas for proteins that are much closer to those of experimental structures than the more extended sidechain regime. The new packing scheme increases changes in non-polar burial for mutants compared to wild-type proteins, but does not substantially improve agreement between ΔG and ΔΔG binding models.

**Conclusion:**

We conclude that solvent accessible area, based on modelled mutant structures, is a poor correlate for ΔΔG upon mutation. A simple volume-based, rather than solvent accessibility-based, model is constructed for ΔG and ΔΔG systems. This shows a more consistent behaviour. We discuss the efficacy of volume, as opposed to area, approaches to describe the energetic consequences of mutations at interfaces. This knowledge can be used to develop simple computational screens for binding in comparative modelled interfaces.

## Background

Macromolecular complexation is key to many biological processes, and has been a subject of experimental study for many decades. The last few years have seen significant advances in high throughput detection of protein-protein interactions, for example with yeast two hybrid [1] and affinity purification methods feeding into analysis by mass spectrometry [2]. These laboratory advances have led to a new area of bioinformatics analysis, interpreting the data in terms of interaction networks, and putting such networks in a biological context [3]. At the same time, structural biology continues to visualise interfaces at atomic resolution [4,5], whilst computational biology addresses whether proteins can be docked into the correct complexes [6,7], and develops models for the prediction of binding affinities [8]. These are fundamental questions, combining the physico-chemical properties of atomic interactions with biological activity.

Docking of two proteins is determined by complementarity of shape and of pairwise interactions within shape-matched patches [9]. Methods for predicting mainchain alteration are still relatively poor, so that successful docking methods have been largely restricted to proteins for which there is little conformational change between the complexed and uncomplexed forms [10]. The issue of sidechain rotameric variation upon complexation can also cause problems [11], necessitating the development of methods to sample sidechain conformers [12]. There are promising advances in the handling of conformational variation, for both sidechains and mainchain, which simulate variation and show that the correct solution can be identified in a cluster of well-packed configurations [13,14].

Computational research in protein docking inevitably overlaps studies that construct models for binding affinities, through the common use of force-fields. One particularly important growth area is the assessment of binding potential for proteins that are homologous with the constituents of characterised complexes i.e. comparative modelling of complexes based on known interfaces [15,16]. Whereas comparative modelling of individual domains based on homology demonstrates the fold for the sequence of interest, albeit with variations that may not be easy to model, similar modelling applied separately to non-covalently linked components must address the question of whether a viable interface is maintained. If effective algorithms can be developed in this area, then structural bioinformatics, combined with structural biology, will be a valuable complement to the high throughput experimental methods for determining protein-protein interactions. The question of what features determine interfacial stability had been extensively studied. A recurring theme is the importance, in many cases, of buried non-polar area [17]. This simple observation, along with the complexity and computational scale of attempts to calculate free energies of binding by simulation, has led to the development of empirical binding models [8]. These involve the separation into terms that each represent some physical feature or combination of features, and which are linearly combined and the relevant weights determined through fitting to a test set of experimental data. Terms reflect properties such as: the hydrophobic effect/buried non-polar surface area; electrostatic interactions; van der Waals interactions; sidechain rotameric entropy; rotational and translation entropy of complexation. The limitations of such models, especially the lack of a simulation component, are counteracted by their potential to detect trends through fitting to experimental data.

There is also widespread interest in modelling the influence of mutations at interfaces, which in biological terms could map for example to the role of single nucleotide polymorphisms in complexes [18]. Binding models have been derived from fitting to measured differences in binding energies for mutant complexes relative to wild-type [19,20]. In the current work, we denote models for binding in complexes as ΔG models, and those for differences (upon mutation) between complexes as ΔΔG models. Since ΔG and ΔΔG models use the same range of terms, apart from cancellation of rotational/translational entropy, it is of interest to compare them. This is the subject of the current study.

An important property of interfaces is the degree to which conformation changes between the complexed and uncomplexed forms. We have restricted our dataset of wild-type proteins to those for which there is no evidence in the literature for mainchain changes, and for mutant proteins the assumption is made that the backbone does not change. With regard to sidechains, we make use of a mean field technique adapted previously in our laboratory [21], from earlier work [22]. With this method the probability of a rotamer is higher where it can fit with a larger number of neighbouring sidechain rotamers, at a given van der Waals tolerance. Thus higher probability values, in this framework, tend to select rotamers with higher solvent accessibility, and conversely lower probability generally maps to lower solvent accessibility. Rotamer prediction is more difficult for sidechains without structural constraints [23], and prediction accuracy reduces in these cases for commonly-used methods such as SCWRL [24]. In the current work we study the effect of differential modelling of interfacial changes, using either the higher solvent accessibility scheme ("HighSA"), or in the lower solvent accessibility ("LowSA") scheme. Comparison with accessible solvent areas in experimental structures suggests that the more compact, LowSA, configurations may give a more appropriate representation.

In addition to the question of how to repack sidechains, there is also the issue of which sidechains to repack. In a minimal scheme for repacking, using the experimental rotamers where possible, wild-type complexes are not repacked at all, and mutations are modelled simply by rotamer selection for the mutated amino acid. Such structural models we denote as "Minimal". In contrast, it is also possible to repack all sidechains for all molecules, so that the packing can (in principle) change upon complexation and/or upon mutation. We label such schemes as "Complete". Thus Minimal-HighSA packed wild-type complexes in ΔG models are simply the structures from the experimentally-determined complexes, and for mutations in ΔΔG models would simply be modelling of the mutated residue with the most solvent accessible rotamer, in the free and complexed states. Complete-LowSA takes low solvent accessibility rotamers, and repacks all sidechains, in both free and uncomplexed states.

Our results show that the importance of buried non-polar surface for ΔG models is not reflected in ΔΔG models, when using HighSA packing (Minimal or Complete). We examine the effect of Complete-LowSA packing, allowing for greater environment-specific relaxation. Although the magnitude of calculated non-polar burial differences between wild-type and mutants is increased, the Complete-LowSA packing does not significantly improve consistency between ΔG and ΔΔG models. A key factor in the inconsistency is that changes in non-polar burial upon mutation can be either positive or negative, in comparison with the experimental ΔΔG values in our dataset that are almost exclusively unfavourable upon mutation. This is true for all packing schemes used, presumably reflecting inaccuracies in predicting conformation upon mutation and/or that structure is more fluid than can be represented by single conformers.

The inconsistency of ΔG and ΔΔG models is substantially reduced when a simple volume-based representation of interfacial changes replaces the area-based description. We discuss this result in the context of comparative modelling for protein-protein interfaces.

## Results and discussion

### Non-polar surface area dominates ΔG binding models (no sidechain repacking)

A set of protein-protein complexes with known structures and binding energies was collated as described in the Methods section. We ascertained that there was no evidence for substantial mainchain conformational change in these complexes through a survey of the literature. Although structures for the uncomplexed components were not used in calculations, in several cases these were used for assessment of conformational change. Table 1 lists the set of wild-type complexes used in the current study, together with their complexation energies.

**Table 1:** Wild-type complexes and binding energies.

**Protein A**	**Protein B**	**ΔG (kJ/mole)**	**PDB**	**Ref**
BPTI	Chymotrypsin	-44.96	1CBW	[49]
Barnase	Barstar	-79.50	1B27	[50]
Subtilisin Carlsberg	OMTKY3	-59.31	1R0R	[51]
Rap1A	Raf1	-35.98	1C1Y	[52]
Ras	Byr2	-38.45	1K8R	[53]
Fv D1.3	Fv E5.2	-45.48	1DVF	[54]
Fv D1.3	HEWL	-45.10	1VFB	[55]
BPTI	Trypsin	-75.16	2PTC	[49]
HyHEL10 Fab	HEWL	-56.21	3HFM	[56]
RalGDS	Ras	-35.15	1LFD	[57]
Subtilisin Carlsberg	Eglin C	-54.76	1CSE	[58]
IM9	Colicin E9	-78.62	1EMV	[59]
HyHEL5 Fab	HEWL	-59.36	1YQV	[60]
SGPB	OMTKY3	-61.45	3SGB	[61]
Ribonuclease Inhibitor	Angiogenin	-87.15	1A4Y	[62]
N9 Neuraminidase	NC10 Fab	-48.50	1NMB	[63]
Subtilisin BPN'	SSI	-61.33	2SIC	[64]
Thrombin	Thrombomodulin	-53.09	1DX5	[65]
Ribonuclease A	Ribonuclease Inhibitor	-76.30	1DFJ	[62]
Kallikrein A	BPTI	-51.83	2KAI	[66]

Protein constituents of the complexes are given, with the following abbreviations: OMTKY3, turkey ovomucoid third domain; HEWL, Hen Egg White Lysozyme; BPTI, Bovine Pancreatic Trypsin Inhibitor; IM9, Immunity Protein 9; SGPB *Streptomyces griseus *protease B; SSI, *Streptomyces *Subtilisin Inhibitor.

**Figure 1:**
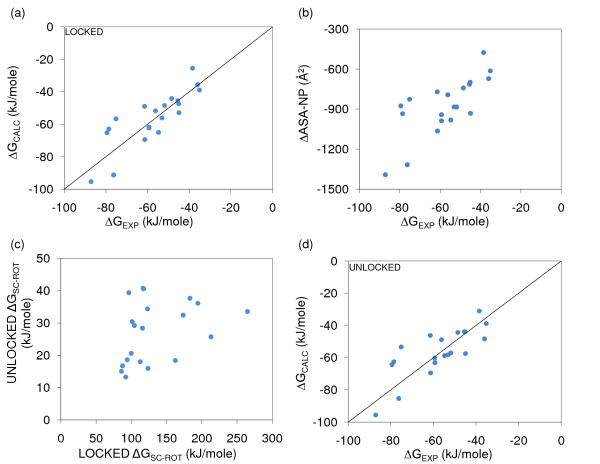
**ΔG models with Locked and Unlocked interfacial sidechains, Minimal-HighSA**. Binding models are fit to wild-type ΔG_EXP_, and Minimal-HighSA implies no repacking for wild-type systems. (a) Model fit with ΔG_SC-ROT _calculated for Locked interfacial sidechains and ΔG_ROT-TRANS _= 0 (R^2 ^= 0.69). (b) Correlation between ΔG_EXP _and Δ(ASA-NP) (R^2 ^= 0.55). (c) Poor correlation (R^2 ^= 0.12) between ΔG_SC-ROT _calculated with Locked and Unlocked interfacial sidechains. (d) Model fit with ΔG_SC-ROT _calculated for Locked interfacial sidechains and ΔG_ROT-TRANS _= 0 (R^2 ^= 0.61). For plots with equivalent y and x quantities, the line y = x is shown.

Figure 1 shows best fit models for the wild-type complexes, setting ΔG_ROT-TRANS _to zero. Table 2 gives the model weights corresponding to Figure 1, and with models calculated for ΔG_ROT-TRANS _= 10 kJ/mole, investigating the range derived from experiment [25]. There is very little difference in models at 0 or 10 kJ/mole for ΔG_ROT-TRANS_, and this is generally true throughout this work. The Methods section describes modelling of ΔG_SC-ROT _according to a Locked scheme (sidechains free in separated components and fixed in the interface), and an Unlocked scheme (free at all points). Comparing Figure 1 panels (a) and (d), and also Table 2, whilst there are some differences between the Locked and Unlocked interfacial sidechain models for ΔG_SC-ROT_, with somewhat better performance for the Locked case, the overall domination by ΔG_ASA-NP _is common. Figure 1(c) shows that Locked ΔG_SC-ROT _values are, as expected, substantially larger than the Unlocked sidechain ΔG_SC-ROT _values, but with little correlation between them. Table 2 indicates that the best fit model uses a negative (unphysical) weight for Unlocked interfacial sidechain ΔG_SC-ROT_. Generally, several of the weights reported in Table 2 are negative. Where these are of small magnitude they will have little contribution to a binding model, and larger magnitude negative weights are likely to result from anti-correlation with other features that have large and positive weights. Table 3 shows correlations between calculated properties, and between calculated properties and experimental binding energies. The correlation shown in Figure 1(b) and the weights in Table 2 make it clear that buried non-polar area dominates the ΔG binding model [17].

**Table 2:** Weights for ASA-based binding models.

**Model type**	**ΔG**	**ΔG**	**ΔG**	**ΔG**	**ΔΔG**	**ΔΔG**	**ΔG**	**ΔG**	**ΔΔG**	**ΔΔG**
	
**ΔG_ROT-TRANS_**	0	10	0	10	-	-	0	0	-	-
**Un/Locked**	L	L	U	U	L	U	L	U	L	U
	
**Packing Scheme**	Minimal-HighSA						Complete-LowSA			
	
**ASA-NP**	0.73	0.79	0.60	0.65	0.07	-0.04	0.58	0.56	-0.31	-0.23
**ASA-P**	-0.04	0.04	-0.08	0.02	-0.73	-0.67	-0.05	-0.11	-0.65	0.04
**IONIS-FDDH**	-0.01	-0.04	0.15	0.13	0.21	0.08	0.10	0.10	0.17	0.16
**IONIS-DESOLV**	0.15	0.09	0.06	0.01	1.29	0.97	0.20	0.19	0.67	0.61
**SC-ROT**	0.12	0.10	-0.22	-0.30	-0.02	-1.61	0.03	-0.02	-0.50	-0.61
**R^2 ^correlation**	0.69	0.64	0.61	0.58	0.61	0.64	0.69	0.68	0.54	0.55
**Figure/Panel**	1a	-	1d	-	2a	2b	6	-	-	-

Weights are either w for ΔG models or w' for ΔΔG models. U/L is Unlocked/Locked interfacial sidechains, applied to SC-ROT.

**Table 3:** Correlations (R) for ΔG model features in wild-type complexes, Minimal-HighSA packing

	**ΔASA-NP**	**ΔASA-P**	**ΔIONIS-FDDH**	**ΔIONIS-DESOLV**	**ΔSC-ROT Locked**	**ΔSC-ROT Unlocked**	**ΔG_EXP_**
**ΔASA-NP**	1						
**ΔASA-P**	0.57	1					
**ΔIONIS-FDDH**	0.31	0.50	1				
**ΔIONIS-DESOLV**	0.55	0.92	0.51	1			
**ΔSC-ROT L**	-0.46	-0.69	-0.25	-0.76	1		
**ΔSC-ROT U**	-0.39	-0.14	0.13	-0.24	0.35	1	
**ΔG_EXP_**	0.74	0.45	0.42	0.54	-0.17	-0.31	1

### Non-polar surface is a minor feature in ΔΔG binding models (minimal sidechain repacking, Minimal-HighSA)

**Figure 2:**
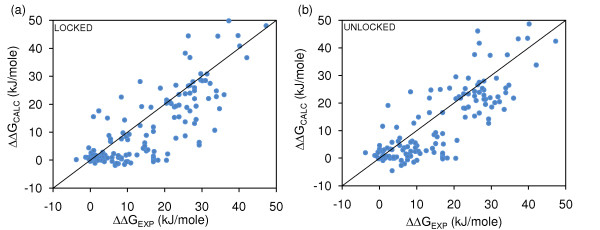
**ΔΔG models for Locked and Unlocked interfacial sidechains, Minimal-HighSA**. Binding models fit to ΔΔG_EXP_, for Minimal-HighSA repacking. (a) ΔG_SC-ROT _calculated for Locked interfacial sidechains (R^2 ^= 0.61). (b) ΔG_SC-ROT _calculated for Unlocked sidechains (R^2 ^= 0.64).

Empirical binding models generally target either complexes or differences between wild-type and mutant complexes, but not both. We are interested in whether ΔG and ΔΔG models show the same relative importance of model features. Figure 2 shows results for ΔΔG models, where the rotation/translation term cancels between wild-type and mutant systems. Again reasonable fits are obtained, although this time the Unlocked interfacial sidechain entropy term gives a slightly better model than the locked term (compare panels (a) and (b) of Figure 2). As for the wild-type calculations, these two terms do not correlate well (not shown). A major difference to ΔG models (Table 2) is that non-polar surface area has a small weight, and a poor correlation with the measured binding data (Table 4). The ΔΔG_ASA-P_, ΔΔG_IONIS-DESOLV _and ΔΔG_SC-ROT _terms correlate to such a degree that individually they can adopt a large and unphysical weighting, with compensation by the other features.

**Table 4:** Correlations (R) for ΔΔG model features (mutant and wild-type complexes differenced), Minimal-HighSA packing

	**ΔΔASA-NP**	**ΔΔASA-P**	**ΔΔIONIS-FDDH**	**ΔΔIONIS-DESOLV**	**ΔΔSC-ROT Locked**	**ΔΔSC-ROT Unlocked**	**ΔΔG_EXP_**
**ΔΔASA-NP**	1						
**ΔΔASA-P**	-0.13	1					
**ΔΔIONIS-FDDH**	-0.06	0.11	1				
**ΔΔIONIS-DESOLV**	-0.22	0.87	0.11	1			
**ΔΔSC-ROT L**	-0.26	0.07	0.01	0.13	1		
**ΔΔSC-ROT U**	0.08	-0.52	-0.35	-0.62	-0.11	1	
**ΔΔG_EXP_**	-0.18	0.55	0.30	0.74	0.29	-0.65	1

### A ΔG binding model fits poorly to measured ΔΔG (minimal sidechain repacking, Minimal-HighSA)

**Figure 3:**
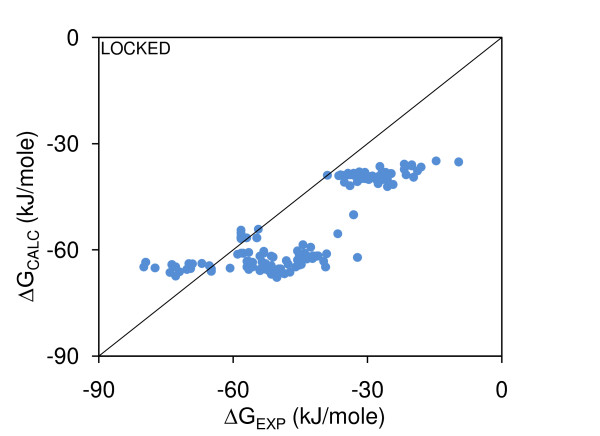
**Wild-type complexes ΔG model applied to mutant complexes, Minimal-HighSA**. The ΔG model fit to wild-type complexes, with Locked interfacial sidechains and ΔG_ROT-TRANS _= 0 applied to mutant complexes, all calculations with Minimal-HighSA packing (R^2 ^= 0.66).

Figure 3 demonstrates discrepancy between ΔG and ΔΔG models. The ΔG model of Figure 1(a) (see also Table 2) has been applied to the mutant complexes, and compared with measured ΔG. Within clusters (representing the mutant sets), the spreads of calculated ΔG are too small to reproduce variation in the measurements. This issue of model incompatibility is not resolved when a single ΔG model is fit to ΔG data for wild-type and mutant complexes (not shown). Some resolution of the discrepancy could be achieved if assessment of buried non-polar area were inaccurate in ΔG or ΔΔG models, and/or if the strategies for mutant selection, and perhaps the systems undergoing mutagenesis, emphasise other features over non-polar properties. The second factor is unlikely to be the whole story, since polar features are down-weighted in the ΔG models. With regard to the first factor, the mean-field packing algorithm has been modified such that sidechain rotamers can be chosen that will tend towards maximal packing with the rest of a protein (LowSA).

### Repacking sidechains with the mean-field algorithm

**Figure 4:**
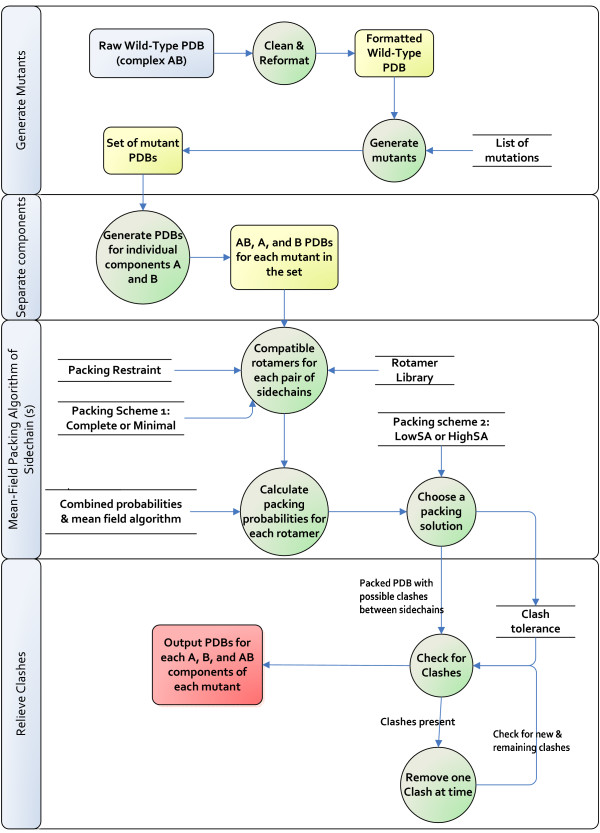
Scheme for generation of mutant complexes and different sidechain packing.

Figure 4 shows the scheme for generation of mutant structures, using the various sidechain packing schemes that are available, Minimal or Complete and HighSA or LowSA. The previous Results sections referred to Minimal-HighSA. Following the observation that buried non-polar ASA dominates ΔG, but not ΔΔG (with Minimal-HighSA binding models), we now consider the effect of using Complete-LowSA repacking. We theorise that in models of mutant structures, particularly those with replacement by alanine, sidechains will relax into the space left by the mutation. Since there may be different constraints on such relaxation in the complexed and uncomplexed states, this could lead to changes in buried area.

**Figure 5:**
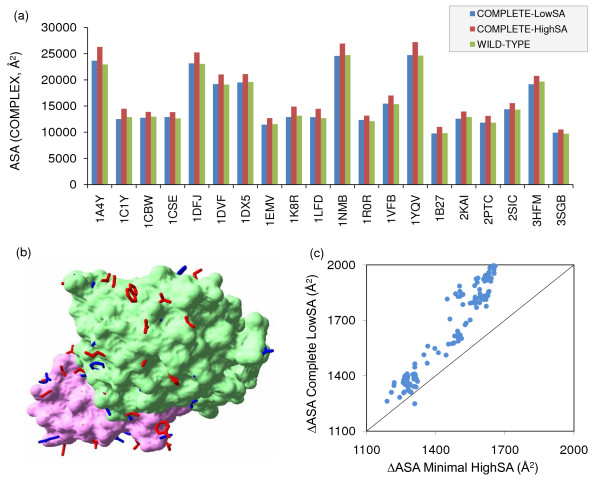
**Complete-HighSA and Complete-LowSA sidechain packing compared**. (a) Total ASA for wild-type complexes, calculated for the experimental structures (wild-type), and repacking of all sidechains in the Complete-HighSA and Complete-LowSA schemes. (b) Molecular surface of experimental structure (1b27 subunits A and D) for barnase (green) – barstar (pink), with Complete-HighSA (red) and Complete-LowSA (blue) repacked sidechains protruding. Drawn with the program Swiss-PdbViewer [48]. (c) Correlation between Δ(ASA) (total buried surface area) in the complexes for Complete-HighSA and Complete-LowSA repacking (R^2 ^= 0.91).

The potential for LowSA prediction is shown with the wild-type complexes in Figure 5(a), where the total ASA values are shown for: experimental structures, Complete-HighSA repacking and Complete-LowSA repacking. LowSA gives an overall result more in accord with experiment. Figure 5(b) shows an example for the barnase-barstar complex (1b27, [26]), where more sidechains packed with Complete-HighSA protrude from the crystal structure molecular surface, than do those packed with Complete-LowSA. In terms of predicted buried ASAs (polar and non-polar combined), Figure 5(c) shows that for the set of mutants used in this study, Complete-LowSA repacking gives consistently more burial than does Complete-HighSA. This result holds when mutant systems are differenced to wild-type, and also whether the mutants are to alanine or other amino acids (not shown). Therefore the ΔΔG_ASA-NP _and ΔΔG_ASA-P _terms are larger (for a given weighting) with Complete-LowSA packing than with Complete-HighSA, and we next examined whether this alters the discrepancy between ΔG and ΔΔG models.

### Combining ΔG and ΔΔG binding models (Complete-LowSA sidechain repacking)

Table 2 lists the best fit models for Complete-LowSA packing applied to the wild-type complexes. The "Complete" packing schemes allow for sidechain relaxation between complexed and uncomplexed forms, including sidechain repacking in the experimentally-determined structure of the complex. Generally sidechains away from the interface will pack similarly in the Complete-LowSA scheme in complexed and uncomplexed forms, therefore cancelling in the binding calculations. The ΔG models for Complete-LowSA are not very different from those for Minimal-HighSA. Models using Locked or Unlocked interfacial sidechains for the rotameric entropy estimation are almost identical for Complete-LowSA. Also, the differences between ΔG_ROT-TRANS _= 0 and ΔG_ROT-TRANS _= 10 calculations are again small (not shown).

**Figure 6:**
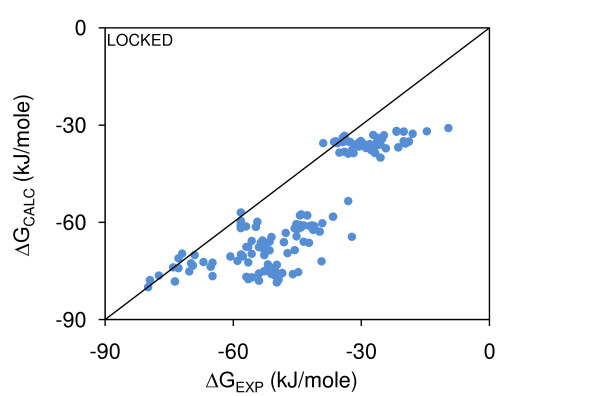
**Wild-type complexes ΔG model applied to mutant complexes, Complete-LowSA**. The ΔG model fit to wild-type complexes, with Locked interfacial sidechains and ΔG_ROT-TRANS _= 0 applied to mutant complexes, all calculations with Complete-LowSA packing (R^2 ^= 0.73).

Figure 6 shows the best-fit Complete-LowSA ΔG binding model, derived for wild-type complexes, applied to ΔG data for mutant complexes (with all molecules repacked in the Complete-LowSA scheme). This is for rotameric entropy estimated from Locked sidechains at the interface, although the low weighting in the model means this will have little influence, and with ΔG_ROT-TRANS _= 0. Figure 6 can be compared directly with Figure 3 (the equivalent Minimal-HighSA calculation), and it can be seen that these plots are qualitatively similar. We had hypothesised that the increase in buried non-polar surface area for Complete-LowSA over Minimal-HighSA models, could lead to greater consistency between ΔG and ΔΔG models. This is not the case.

### Recalculating ASA at lower solvent probe radius

**Figure 7:**
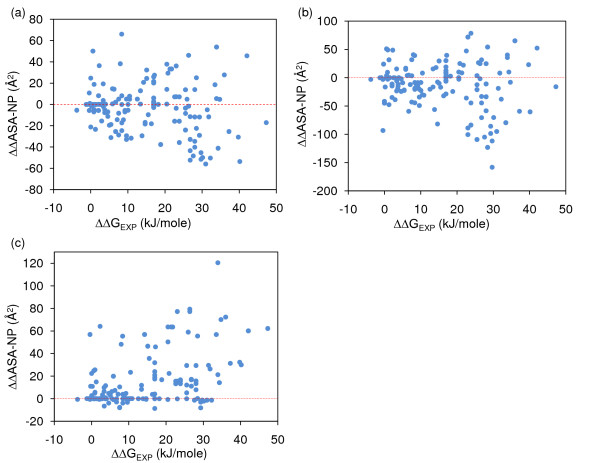
**ΔΔ(ASA-NP) does not correlate with ΔΔG_EXP_, and is of variable sign**. (a) ΔΔ(ASA-NP) calculated with Minimal-HighSA packing does not correlate with ΔΔG_EXP _(R^2 ^= 0.00), and can be positive or negative (red line drawn at ΔΔ(ASA-NP) = 0). (b) Qualitatively similar behaviour to that for panel (a) is seen for Complete-LowSA repacking (R^2 ^= 0.02). (c) Reducing the solvent probe radius from 1.4 Å to 0.5 Å gives mostly uniform sign of ΔΔ(ASA-NP), but still poor correlation with ΔΔG_EXP _(R^2 ^= 0.18).

Since ΔG_ASA-NP _drives agreement for ΔG models, but not for ΔΔG models (whichever sidechain packing is used), we looked more closely at this property in the mutant interfaces. From Figure 7(a), (b) it is apparent that both Minimal-HighSA and Complete-LowSA schemes give a spread of ΔΔ(ASA-NP) values around zero, albeit with larger magnitude in the Complete-LowSA scheme. Since ΔΔG_EXP _is almost exclusively of one sign, it is clear that ΔΔG_ASA-NP _will be down-weighted in a best fit linear model. Figure 7(c) shows that the dual sign spread of ΔΔ(ASA-NP) is largely negated when the solvent probe radius is reduced from 1.4 Å to 0.5 Å, but the correlation with ΔΔG_EXP _remains poor. These results indicate that ΔΔG_ASA-NP_, with the current repacking schemes, is not an effective feature with which to understand ΔΔG binding models. Visual inspection (not shown) reveals that relatively large non-polar surfaces may be revealed upon mutation, and whether or not these are solvent accessible depends on the fine detail of packing differences between a complex and its components. Whereas ASA-NP is the most prominent feature in ΔG models, either it is not an appropriate description for interfacial relaxation in response to mutation, or current modelling of interfacial relaxation is insufficient.

### Volume-based solvation

We reasoned that a volume-based solvation function would be less sensitive to the details of packing changes than a solvent accessible area-based feature. This is consistent with the more prominent role played by ΔG_IONIS-DESOLV _in ΔΔG models, as opposed to ΔG models. Solvation shell models have been investigated previously in the context of solute/protein structure and stabilisation [27-29]. Here we develop simple properties to replace Δ/ΔΔG_ASA-NP _and Δ/ΔΔG_ASA-P _in the binding models, in which solvation shell volumes are calculated for each atom, on a grid. The volumes cover a shell of 2.8 Å thickness around atomic van der Waals radii, and are assigned polar or non-polar according to atom type. Then Δ (complexation) and ΔΔ (mutant to wild-type and complexation differences) are calculated for G_VOL-NP _and G_VOL-P_. This model affords a rapid calculation, and volume-based features give the uniform sign behaviour expected for mutants that generally involve reduction of sidechain size (not shown), unlike the distribution of ΔΔ(ASA-NP) (Figure 7(a)).

**Figure 8:**
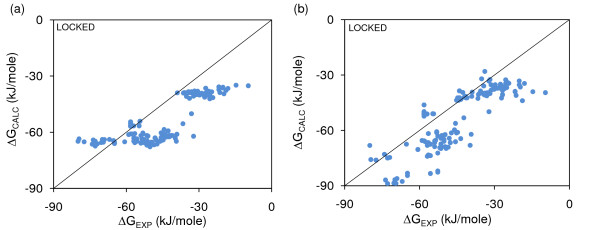
**ASA-based and volume-based solvation ΔG models compared**. Calculations for ΔG of mutant complexes, using ΔG models fit to wild-type complex data, with ΔG_SC-ROT _calculated for Locked interfacial sidechains, Minimal-HighSA repacking and ΔG_ROT-TRANS _= 0. (a) ASA-based polar and non-polar solvation terms (R^2 ^= 0.66). (b) Volume-based solvation (R^2 ^= 0.68). Although there is little difference in R^2^, the spread in ΔG_CALC _within mutant systems (i.e. based on the same wild-type) is larger with volume calculations.

Figure 8 compares Minimal-HighSA models for area and volume-based features (Table 5), where models have been fit to wild-type ΔG data and calculated and plotted for the mutant ΔGs. The mutant data in both panels show an unequal distribution around the ΔG_CALC _= ΔG_EXP _line, since the 4 underlying systems are a subset of the 20 wild-type complexes used to generate the area and volume-based ΔG_CALC _models. A relatively flat spread of mutant clusters for the area-based model is much less apparent with the volume-based model, indicating a more consistent modelling of variation within these clusters, although R^2 ^is not much different between the two panels of Figure 8. Further evidence of the effectiveness of volume-based modelling is seen when comparing equivalent ΔG and ΔΔG models in Tables 2 and 5. For Minimal-HighSA and Locked interfacial sidechains, feature weights change entirely between ASA-based ΔG and ΔΔG models (e.g. with the non-polar ASA term going from dominating to being insignificant). For volume-based models, it is actually the polar term that is more important, but this is maintained on moving from ΔG to ΔΔG. Weight variations for ΔG and ΔΔG models with Complete-LowSA repacking are more complex.

**Table 5:** Weights for volume-based binding models.

**Model type**	**ΔG**	**ΔG**	**ΔΔG**	**ΔΔG**	**ΔG**	**ΔG**	**ΔΔG**	**ΔΔG**
	
**ΔG_ROT-TRANS_**	0	0	-	-	0	0	-	-
**Un/Locked**	L	U	L	U	L	U	L	U
	
**Packing Scheme**	Minimal-HighSA				Complete-LowSA			
	
**VOL-NP**	-0.12	-0.11	-0.31	-0.28	0.20	0.24	0.04	0.02
**VOL-P**	1.00	0.85	1.79	1.45	-0.03	-0.08	0.55	0.61
**IONIS-FDDH**	0.44	0.43	-0.01	-0.05	0.19	0.18	0.28	0.29
**IONIS-DESOLV**	-0.19	0.11	0.26	0.23	0.17	0.26	0.30	0.35
**SC-ROT**	-0.07	-0.49	-0.01	-1.04	-0.06	0.08	-0.24	-0.29
**R^2 ^correlation**	0.46	0.50	0.62	0.64	0.64	0.66	0.64	0.66

Weights are w for ΔG model, w' for ΔΔG models. U/L is Unlocked/Locked interfacial sidechains, applied to SC-ROT.

Looking at calculations with Minimal-HighSA repacking, it appears that even simple volume calculations are capturing common properties in the ΔG and ΔΔG processes, a behaviour that has largely defeated the ASA-based calculations. The volume-based terms are particularly rapid to calculate, and could provide the basis for simple, low resolution, computational screens of interface viability. Table 6 shows ΔG and ΔΔG models derived using only two volume-based solvation terms. The R^2 ^values for Minimal-HighSA models with just two features are close to those of the best fits for the more extensive Minimal-HighSA models given in Table 5.

**Table 6:** Weights for binding models with only volume-based features.

**Model type**	**ΔG**	**ΔΔG**	**ΔG**	**ΔΔG**
	
**ΔG_ROT-TRANS_**	0	-	0	-
	
	Minimal-HighSA		Complete-LowSA	
	
**VOL-NP**	-0.13	-0.40	0.18	0.12
**VOL-P**	0.94	2.30	0.25	0.91
**R^2 ^correlation**	0.43	0.61	0.43	0.49

Weights are w for ΔG models, w' for ΔΔG models.

Our investigation of simple, empirical models for binding that can generalise between ΔG and ΔΔG applications, contrasts with interface-specific methods using quantitative structure-activity relationships that highlight residues of particular importance [31]. In practice both approaches have the same overall aim, i.e. predictive capability for protein-protein interactions; and the same constraints, with sets of correlated features (Tables 3 and 4) and an imprecise understanding of the underlying conformational and energetic framework. Our observation that volume-based terms perform better than area-based terms in ΔΔG models highlights this latter problem. It is possible that modified area-based terms, for example with conformational sampling and weighting, could improve performance.

## Conclusion

We find that binding models, using minimal sidechain repacking and ASA-based solvation terms, are quite different depending on whether they are fit to data for ΔG (wild-type complexes) or ΔΔG (modelled mutant complexes differenced to wild-type complexes). Whereas buried non-polar area dominates the ΔG model (Figure 1, Table 2), consistent with previous work [17], other interactions assume much greater importance for the mutant complexes (Figure 2, Table 2), and the ΔG binding model does not reproduce the spread of ΔG_EXP _within mutant sets (Figure 3).

Investigating whether different sidechain repacking could alter this discrepancy, a scheme for packing sidechains towards protein structure has been derived from a mean-field framework (Figure 4). This method, which we label Complete-LowSA since all sidechains (mutated or not) are repacked, is promising in terms of a better agreement with total ASA for experimental complexes, and in giving larger buried surface areas upon complexation than the Minimal-HighSA scheme (Figure 5). However, this does not lead to significant increase in the importance of non-polar buried area in best fit ΔΔG models, and there remains a discrepancy between ΔG and ΔΔG models (Figure 6). Further analysis of ΔΔ(ASA-NP) revealed a spread around zero that mitigates against fitting to ΔΔG_EXP_, which is predominantly single sign (Figure 7). These results indicate that either non-polar buried area is not important for ΔΔG modelling, which would be surprising given its role in ΔG modelling, or that we are not capturing the complexity of sidechain (and potentially mainchain) conformational rearrangement upon mutation.

Of the features studied, ionisable group-based electrostatics contributes relatively little in best fits to experimental data, for both ΔG and ΔΔG models. Clearly there will be instances where ionisable groups will contribute substantially to interfacial energetics, and mediate processes such as a physiological pH-dependence of binding. In general though, studies of optimal predictors of interfacial propensity show that net charge is (in relative terms) excluded, part of an overall tendency for ease of desolvation at interfaces [30]. For most ΔG and ΔΔG models, sidechain rotameric entropy plays a relatively small part (Table 2), and where the weight is large may be partly due to correlations with other properties (Tables 3, 4). In ΔΔG models with ASA-based solvation, ΔΔG_ASA-P _and ΔΔG_IONIS-DESOLV _assume more importance. Polar solvation may be reflecting overall burial change in the interface upon mutation, rather than indicative of particular favourable polar interactions. The effects of mutations on polar area relative to non-polar area are somewhat different. The polar area equivalent to the non-polar area data plotted in Figure 7(a) (Minimal-HighSA) is qualitatively similar, whereas the polar area equivalent to Figure 7(b) (Complete-LowSA) is qualitatively different, being largely of a single sign (not shown).

Empirical desolvation for ionisable groups, describing an estimate of the entropy of water molecule liberation, is a simple volume-based term. We therefore tested the capacity for volume-based terms in general to account for solvation changes in ΔΔG models, in place of area-based terms. The volume solvation features assume importance in all models, reducing the discrepancy between ΔG and ΔΔG models (Figure 8). Volume-based models are part of the molecular mechanics force-fields in some applications [20,29], and ΔΔG models with volume-based solvation terms have been used, in part for faster calculation [19,20]. In the current work, we show that even a very simple solvation shell model is effective in improving consistency of ΔG and ΔΔG models. Surface area-based modelling for ΔΔG fails since ASA is particularly sensitive to relatively small conformational changes, even for single site mutations. In contrast volume based models are less sensitive, and a simple binding model for ΔG and ΔΔG can be constructed with just polar and non-polar volume-based features. This could be useful in computational assessment of the validity of comparative modelled interfaces, and our simple implementation is made available for such analyses.

## Methods

### Binding data and protein structures

Protein complexes were selected where the literature and structural databases give no evidence of major conformational change in the mainchain upon complexation, and for which measured ΔG is available. The literature e.g. [31] and various databases were used to search for binding energies associated with complexation, the alanine scanning energetics database (ASEdb, [32]), the protein-protein interactions thermodynamic database (PINT, [33]), and the ProTherm thermodynamic database for proteins and mutants [34]. Imposition of limited conformational change led to the inclusion of just 20 complexes, which are listed in Table 1 with their measured binding energies. In cases where binding data are recorded for multiple conditions, that most closely corresponding to our calculation conditions of 300 K, 0.15 M ionic strength, pH 7, were chosen. The data in Table 1 were used to construct ΔG binding models. In calculations, only structure files (from the PDB, [4]) for the complex were used. All components (free proteins and mutated proteins) were derived from these coordinates.

For mutant data in ΔΔG model fitting, 4 of the systems used in ΔG modelling were included. The restriction of no major mainchain changes was therefore carried over to the mutants ΔΔG analysis. These systems, and the number of mutants associated with each, are: barnase-barstar, 64 mutants; chymotrypsin-BPTI, 15 mutants; ralGDS-ras, 49 mutants; trypsin-BPTI, 15 mutants. Where mutants are solely changes to alanine, no repacking is required in the Minimal-HighSA or Minimal-LowSA schemes. Complete-HighSA and Complete-LowSA schemes will repack all sidechains.

### Binding model

We employed an empirical binding model with contributions from the rigid body rotational and translational entropy of complexation (ΔG_ROT-TRANS_), buried non-polar surface area (ΔG_ASA-NP_), buried polar surface area (ΔG_ASA-P_), ionisable group charge interactions (ΔG_IONIS-FDDH_), a term to approximate the free energy of water molecule liberation upon ionisable group burial (ΔG_IONIS-DESOLV_), and sidechain rotameric entropy (ΔG_SC-ROT_). Thus;

ΔG_CALC _= ΔG_ROT-TRANS _+ w_ASA-NP_ΔG_ASA-NP _+ w_ASA-P_ΔG_ASA-P _+ w_IONIS-FDDH_ΔG_IONIS-FDDH _+ w_IONIS-DESOLV_ΔG_IONIS-DESOLV _+ w_SC-ROT_ΔG_SC-ROT_

where the various pre-multipliers (w) are the weights to be adjusted in model fitting to experimental data. More detail follows for the individual terms.

The rotational and translational entropy change associated with complexation of two rigid bodies has been studied experimentally and theoretically [25,35,36]. Measurements suggest that an entropy penalty corresponding to a free energy in the range of 0–10 kJ per mole of interacting species at 300 K is appropriate [25]. This is a relatively small range compared with measured ΔG values (Table 1). Other computational work has set ΔG_ROT-TRANS _to zero [8]. We examine ΔG_ROT-TRANS _at either 0 or 10 kJ/mole for each binding model, to test the sensitivity of the model to this term. Models calculated at these ΔG_ROT-TRANS _values, for a given set of conditions, are very similar (Table 2), so that the precise value is not a major consideration in this study. We are neglecting the entropic term associated with any changes in vibrational modes upon complexation [37].

Non-polar and polar solvent accessible surface areas are calculated with the program SACALC, developed in our laboratory. Areas are then differenced between a complex and the sum of its components, and multiplied by a factor of 0.1 kJ/mole/Å^2^. This factor is within the range generally assumed to represent the energetics of hydrophobic solvation in empirical modelling [38,39]. The precise value is not critical, since linear fitting will scale surface area terms via the pre-multipliers. The same factor of 0.1 has been used for polar surface area, and we include this feature in part to allow for some approximate recognition of hydrogen-bonding potential. Detailed assessment of pairwise hydrogen bonds, outside of the context of ionisable groups, is not included. Additionally, explicit van der Waals interactions are excluded. Both hydrogen bond and van der Waals interactions, with a strong distance-dependence over fractions of an Å, would be more appropriate if energy minimisation were carried out subsequent to sidechain repacking from a discrete rotamer library. We chose to develop binding models without energy minimisation, and this relatively simple approach gives a broad insight into differences between ΔG and ΔΔG models. Both the non-polar and polar area terms, as defined, are favourable for complexation when weights are positive.

Ionisable group interactions were derived from the pH-dependence of electrostatic energy, with addition of a constant of integration at an extreme pH [40]. The pH-dependent electrostatics were calculated with in-house programs using the FD/DH method that combines Finite Difference Poisson-Boltzmann (FDPB, [41]) and Debye-Hûckel (DH) interaction schemes [42]. Monte Carlo sampling [43] was used to derive pKas [44] and the ionisable charge distribution, from which electrostatic energy is obtained [45]. Ionisable group energies are included with the ΔG_IONIS-FDDH _term, and may be either favourable or unfavourable for complexation.

We have previously introduced an empirical term to account for the entropy of water liberation upon ionisable group burial, using a comparison of calculated and measured pKas [46]. This analysis looked at hydration differences between ionised and neutral states. The significance of this empirical parameter in pKa calculations was generally small, which was rationalised in terms of little overall difference between water ordering in the ionised and neutral states. Cysteine was an exception, consistent with relatively little charge separation and reduced hydrogen-bonding potential in the neutral state. The ΔG_IONIS-DESOLV _term in the current study models change in burial of ionisable groups upon complexation, rather than ionised/neutral form differences. In order to model the entropy associated with hydration shell liberation (desolvation) for ionisable groups, we used the derived cysteine value from our previous work, since in this case the neutral form has relatively little charge separation. Then ΔG_IONIS-DESOLV _is this complete hydration shell value multiplied by the change in hydration shell volume (calculated from FDPB grids) upon complexation, with no other variation between ionisable group types. As with other terms, the weight derived by fitting to experimental data will indicate relative importance. With liberation of solvating water upon complexation, this term is expected to be favourable for binding.

Finally in the binding scheme is modelling of free energy changes due to alteration of sidechain rotameric entropy upon complexation, ΔG_SC-ROT_. We use a method based on mean-field packing of sidechains, with derived probabilities (p) for rotamers based on packing opportunities [21,22]. Sidechain rotameric entropy for each amino acid is the sum over p*ln(p) for rotamers. Values are summed over amino acids in a protein, differenced between free and complexed states, and multiplied by RT = 2.5 kJ/mole to give a free energy contribution, prior to weighting by the pre-multiplier. Changes in sidechain rotameric entropy are expected to be unfavourable for complexation (restriction of sidechains).

We used two variations of the sidechain rotamer term. In the first, rotamer packing differences upon complexation are calculated as described, with interfacial sidechains free to explore different packings, even though energy calculation for other terms is based on a single conformer. This variation is termed "Unlocked". In the second, interfacial sidechains (in the complex) are modelled as fixed in the conformation used for other components of the energy calculation. Entropy changes will therefore be larger for the second case, which is described as "Locked". These differences are discussed in the Results section, although generally the impact of the sidechain rotameric entropy was low in the models, whichever scheme was used for interfacial flexibility.

### Binding differences model

We simply difference the binding model between wild-type and mutant systems (ΔΔG_EXP _= ΔG_EXP _[MUT] - ΔG_EXP _[WT]), with the rigid-body rotation and translation term cancelling. A prime has been added to the weights, demonstrating that we are allowing different fits for ΔG and ΔΔG models.

ΔΔG_CALC _= w'_ASA-NP_ΔΔG_ASA-NP _+ w'_ASA-P_ΔΔG_ASA-P _+ w'_IONIS-FDDH_ΔΔG_IONIS-FDDH _+ w'_IONIS = DESOLV_ΔΔG_IONIS-DESOLV _+ w'_SC-ROT_ΔΔG_SC-ROT_

### Model fitting

Multiple linear regression was used to determine the best fit ΔG_CALC _and ΔΔG_CALC _models to experiment. This regression was performed with the built-in least squares function of the GNU Regression, Econometrics and Time-series Library package (GRETL, [47]). The Solver function in Microsoft Excel was also used to carry out linear regression. Weights are expected to be positive to make physical sense, other than for ΔG_IONIS-FDDH_, which could be of either sign with attractive or repulsive interactions to the fore.

### Sidechain repacking

Starting with an experimental structure for a wild-type complex, there are questions of conformational change and repacking for the uncomplexed components and mutated proteins. Data selection should have eliminated systems with large-scale mainchain conformational changes, but sidechain rearrangement remains an issue.

The basis of our methodology is a mean-field program developed [21] from earlier work [22]. This uses pairwise packing of rotamers to derive probabilities for rotamers within a sidechain, according to an allowed van der Waals tolerance in the packing. Higher rotamer probability means coexistence with a larger number of neighbouring sidechain rotamers, and larger solvent accessibility (HighSA). Conversely, the lower (but non-zero) probability rotamers will tend to have lower solvent accessibility (LowSA). Both of these packing schemes/rotamer selections are used in our studies of ΔG and ΔΔG models. Another question for sidechain repacking is whether to remain as close as possible to experimental (complex) structure (Minimal repacking), or whether to allow sidechain relaxations in response to complex separation and mutation (Complete repacking).

### Volume-based solvation

A simple volume-based solvation function was used to replace the ASA-based analysis at some points in the work. Thus, w_VOL-NP_ΔG_VOL-NP _+ w_VOL-P_ΔG_VOL-P _was swapped into the ΔG_CALC _equation in place of w_ASA-NP_ΔG_ASA-NP _+ w_ASA-P_ΔG_ASA-P_, with the analogous weighted terms also for the ΔΔG_CALC _binding model. These volume solvation terms are based on grid calculations of volumes around non-hydrogen atoms that are filled or unfilled by neighbouring atoms. Non-polar atoms and radii (Å) are C:2.0 and S:1.9. Polar atoms and radii are O:1.5 and N:1.8. Volumes for each atom are calculated with a 0.5 Å spaced grid and a shell of thickness 2.8 Å beyond the atomic radius. These calculations give numbers of grid points that are not covered by neighbouring atoms. In order to put the numbers onto a scale roughly equating to that for ASA-based terms, a multiplicative factor applied to the number of grid points in the solvation shell of a C atom was equated with the ASA energy for the same, unoccluded, atom. This multiplicative factor is 0.0042.

## Availability and requirements

Project name: Intcalc

Project home page: 

The software used in this study is also available for download from: 

Operating system(s): Linux

Programming language: Perl, Fortran

License: GNU GPL

No additional restrictions for non-academic users.

## Abbreviations

ASA: Accessible Surface Area; PDB: Protein Data Bank; PINT: Protein-protein Interactions Database; ASEdb: Alanine Scanning Energetics database; BPTI: Bovine Pancreatic Trypsin Inhibitor; FDPB: Finite Difference Poisson-Boltzmann; DH: Debye-Hückel; FD/DH: Finite Difference/Debye-Hückel; GRETL: GNU Regression: Econometrics and Time-series Library.

## Authors' contributions

SB and JW conceived the study, interpreted the data and wrote the final manuscript together. SB and JW both contributed source code, whilst dataset creation and implementation of the computational analyses were due to SB.
